# HIC2 regulates isoform switching during maturation of the cardiovascular system

**DOI:** 10.1016/j.yjmcc.2017.10.007

**Published:** 2018-01

**Authors:** Iain M. Dykes, Kelly Lammerts van Bueren, Peter J. Scambler

**Affiliations:** a Institute of Child Health, University College London, 30 Guilford St, London WC1N 1EH, United Kingdom; b Translational Health Sciences, Bristol Medical School, University of Bristol, Bristol Royal Infirmary, Upper Maudlin St, Bristol BS2 8HW, United Kingdom

**Keywords:** Congenital heart disease, Embryonic development, Haemoglobin, Troponin, Creatine kinase, Myoglobin

## Abstract

Physiological changes during embryonic development are associated with changes in the isoform expression of both myocyte sarcomeric proteins and of erythrocyte haemoglobins. Cell type-specific isoform expression of these genes also occurs. Although these changes appear to be coordinated, it is unclear how changes in these disparate cell types may be linked. The transcription factor *Hic2* is required for normal cardiac development and the mutant is embryonic lethal. *Hic2* embryos exhibit precocious expression of the definitive-lineage haemoglobin *Hbb-bt* in circulating primitive erythrocytes and of foetal isoforms of cardiomyocyte genes (creatine kinase, *Ckm*, and eukaryotic elongation factor *Eef1a2*) as well as ectopic cardiac expression of fast-twitch skeletal muscle troponin isoforms. We propose that HIC2 regulates a switching event within both the contractile machinery of cardiomyocytes and the oxygen carrying systems during the developmental period where demands on cardiac loading change rapidly.

## Highlights

• HIC2 regulates developmental and cell type-specific isoform switching.
• The mouse mutant exhibits precocious embryonic expression of foetal genes.
• Definitive-lineage haemoglobins are expressed in primitive-lineage erythrocytes.
• Mature isoforms of cardiac genes are precociously expressed.
• Skeletal muscle troponin isoforms are expressed in the heart.

## 1 Introduction

The transition from embryonic to foetal development is associated with major changes in physiology. Increased cardiac loading as a result of the gain in size requires improved cardiomyocyte performance. The heart rate increases from 125 bpm to 194 bpm between E10.5 and E14.5 [1]. Cardiomyocytes, in common with other muscle cell types, are highly plastic and are able to adapt to meet this demand by altering the expression of sarcomeric proteins, metabolic enzymes and other proteins [2], [3]. This increased workload requires an increased supply of oxygen and this is met by changes in haemoglobin expression within circulating erythrocytes [4] and upregulation of myoglobin in cardiomyocytes [5]. These changes are mediated by a coordinated transition from an embryonic to a foetal gene expression programme in both cardiomyocytes and in erythrocytes.

Sarcomeric proteins such as myosin, actin, myomesin and troponin exist in multiple isoforms, each with distinct physiological properties, and these may be exploited by the embryo to fine tune performance [6], [7], [8] . Multiple isoforms of these proteins appear to have evolved by duplication of a common ancestral gene, followed by a process of divergence and adaptation [9], [10]. This has led to great diversity. For example, there are 10 isoforms of myosin heavy chain [11] and three isoforms of troponin T [12]. Cardiomyocytes, smooth muscle, fast skeletal muscle and slow skeletal muscle exhibit muscle-specific isoform expression; these isoforms are each adapted to a specific function and are non-redundant [13], [14]. In addition, each muscle type exhibits maturational changes in isoform expression, an adaptation to changing demands [7], [15]. Similarly, a number of isoforms of both alpha and beta haemoglobins have evolved by gene duplication, these exhibit distinct affinities for oxygen and are expressed within circulating erythrocytes in a developmentally regulated sequence [4], [16], [17]. Expression of the cardiomyocyte globin, myoglobin, is initiated in the foetal period [5]. Maturational isoform switching is also seen in metabolic enzymes such as creatine kinase, which supplies energy to muscle, and exists as two isoforms expressed in a developmentally regulated sequence [18], [19]. Pathological conditions leading to hypoxia such as ischaemia, heart failure and atrial fibrillation, can result in a recapitulation of the cardiomyocyte foetal or embryonic gene expression programme [20], [21], [22].

HIC2 is a transcription factor related to the tumour suppressor HIC1 [23], required for normal cardiac development and lost in distal variants of 22q11 Deletion Syndrome [24]. Mice heterozygous for *Hic2* have a ventricular septal defect and exhibit peri-natal lethality [24]. Homozygous loss of function mutants, in contrast, exhibit early embryonic lethality [24], occurring before septation of the heart begins. In an effort to understand the cause of this lethality, we uncovered evidence to suggest that HIC2 may play a role in the regulation of isoform expression in both cardiomyocytes and primitive erythrocytes. We show that HIC2 acts to suppress expression of foetal isoforms, which are normally turned on at a time when *Hic2* expression is decreasing. In the absence of *Hic2*, foetal genes are precociously expressed in both cell types and lineage specific troponin expression is disrupted.

## 2 Results

### 2.1 *Hic2* loss results in developmental delay and early embryonic lethality

**Fig. 1:**
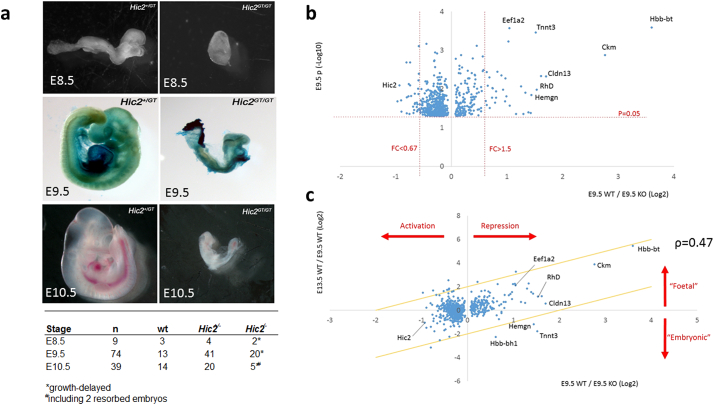
*Hic2* regulates the timing of gene expression in the heart and blood. a. Wholemount images of wildtype (left) and *Hic2^GT/GT^* embryos (right), E9.5 embryos are stained with X-Gal to show expression of the genetrap cassette from the *Hic2* locus. Mutants are delayed by approximately one day of development and are small in size. Embryos are recovered at the Mendelian ratio until day E9.5 but show lethality after this age (Table). b. The graph shows genes changed in a microarray analysis of the *Mesp1^Cre^ Hic2* conditional mutant at E9.5. Fold change (log2 scale) is plotted against *p*-value (− log10 scale). c. The change in expression over developmental time (change between E13.5 and E9.5 in the wildtype condition) is plotted against change in the *Hic2* conditional mutant at E9.5 (E9.5 WT/KO). Genes that are expressed more strongly at E13.5 than at E9.5 may be considered foetal genes while those expressed more strongly at E9.5 may be considered as embryonic. Yellow lines represent a 4 fold change. ρ indicates Pearson's Correlation coefficient calculated from log-transformed values.

Examination of *Hic2^GT/GT^* loss of function mutants revealed that embryonic developmental is slower in these embryos relative to littermate controls. We found that mutant embryos harvested at day E9.5 appeared younger than littermates and exhibited a comparable anatomy to wildtype embryos harvested at E8.5 (Fig. 1a). This delay could not be explained by reduced proliferation or increased apoptosis because no differences were observed in the number of cells positive for either phosphorylated histone H3 and cleaved caspase 3 (data not shown). Embryos also exhibited early embryonic lethality, with few mutant embryos recovered after E9.5 (Fig. 1a).

### 2.2 Foetal genes are precociously expressed in the *Hic2* mutant

Early lethality and reduced size made further analysis of loss-of-function mutants impossible. We therefore turned to a milder, conditional mutant. *Mesp1^Cre^* drives deletion in both cardiac and extra-cardiac derivatives of the anterior mesoderm, the latter including a subset of the haematopoietic system. *Hic2^FL/FL^; Mesp1^Cre/+^* embryos exhibit only a partially penetrant lethality (26% at E13.5), which occurs later in gestation than in Hic2^GT/GT^ embryos [24].

Gene expression analysis was performed in the *Hic2^FL/FL^; Mesp1^Cre/+^* embryo at E9.5 on the isolated heart tube. The samples consisted of cardiovascular tissue together with the blood contained within it. Erythrocytes in circulation at E9.5 are largely nucleated, primitive-lineage erythrocytes which carry mRNA. 62 genes showed > 1.5 fold change (Fig. 1b). The largest changes were seen in upregulated genes (max change 12.1) with only modest changes in downregulated genes (> 0.51). The most changed genes were found to be the beta haemoglobin, *Hbb-bt* (+ 12.1) and the creatine kinase enzyme isoform *Ckm* (+ 6.78). Strikingly, these are both genes whose expression is normally initiated later in development during the foetal period. We hypothesised that loss of *Hic2* may result in precocious expression of a foetal gene expression programme. To test this, we compared these data to a dataset we have previously published derived from the wildtype E13.5 embryo [24] . We plotted the ratio of expression in the wildtype embryo at E13.5 relative to E9.5, against the change in expression in the E9.5 mutant relative to E9.5 wildtype (Fig. 1c). Many genes which show an increase in the *Hic2* mutant at E9.5, suggesting *Hic2* repression, were seen to be more highly expressed later in development (Fig. 1c). Genes showing a decrease in the mutant, which are activated downstream of *Hic2*, did not show strong changes in the mutant, but broadly speaking were more strongly expressed in the embryo than in the foetus.

This analysis raises the intriguing possibility that HIC2 may function to repress expression of foetal genes in order to maintain the heart and circulatory system in an embryonic state.

### 2.3 HIC2 regulates haemoglobin isoform switching

The most changed gene on the microarray was the beta haemoglobin, *Hbb-bt*. Haemoglobin is a tetramer consisting of two alpha and two beta haemoglobin molecules, several isoforms of each exist in the genome with distinct physiological properties and these are classified as either embryonic or definitive, based on the timing of expression [4]. Primitive lineage erythrocytes are derived from MESP1 + precursors in the yolk-sac [25], and express the embryonic haemoglobins, *Hbb-bh* and *Hbb-y*. At day E11.5 the major site of erythropoiesis moves from the yolk sac to the liver, and the resulting liver-derived definitive lineage expresses only the definitive haemoglobins, *Hbb-bt* and *Hbb-bs* [4]. These latter genes are derived from independent loci but have an identical sequence and thus are indistinguishable in either microarray or qPCR assays. Thus, we observe expression of definitive haemoglobins (*Hbb-bt* and/or *Hbb-bs*) at a time when only primitive erythrocytes are in circulation, indicating dysregulation of gene expression in these cells.

**Fig. 2:**
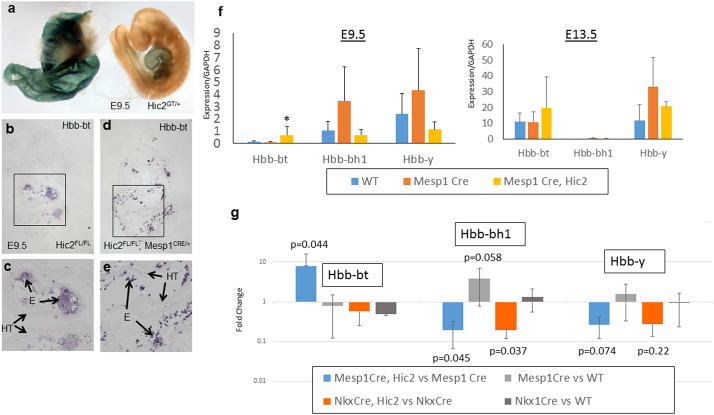
HIC2 represses foetal haemoglobin expression in primitive erythrocytes. a. X-gal staining of an E9.5 *Hic2^GT/+^* embryo reveals strong reporter expression in the yolk sac (left) as well as in the heart tube (right). b–e. *In situ* hybridisation to show expression of *Hbb-bt* at E9.5 in the wildtype (b,c) and mutant (d,e) embryo. Lower panels show an enlarged view of the boxed area above. E = erythrocyte; HT = heart tube. f–g. RT-qPCR analysis of gene expression in pooled heart samples. f and g show absolute expression (normalised to GAPDH) at E9.5 and E13.5 respectively while g shows the fold change between the genotypes indicated at E9.5. *P* values indicate results of a one tailed *t*-test.

To begin to explore this phenomenon we first asked whether *Hic2* is expressed in primitive erythrocytes or their precursors. X-gal staining of *Hic2^GT/+^* embryos revealed strong reporter gene expression in the yolk sac at E9.5, in addition to the previously reported heart expression (Fig. 2a). Thus, *Hic2* is expressed at the site of primitive lineage erythropoiesis. *Mesp1* is known to be expressed in yolk sac blood islands and primitive erythrocytes are derived from MESP1 positive precursors [25], therefore it is reasonable to suggest that *Mesp1^Cre^* could delete *Hic2* in primitive erythrocyte precursors.

We next used *in situ* hybridisation to confirm the change in expression of *Hbb-bt* seen on the microarray. Staining of wildtype embryos revealed a low level of expression restricted to erythrocytes, which are located largely within the heart tube (Fig. 2b, c). This result is consistent with reports that primitive lineage erythrocytes express low levels of definitive haemoglobins [16], [17]. In the *Mesp1^Cre^* conditional mutant we see strong upregulation of expression in erythrocytes (Fig. 2d, e) confirming the array results.

To quantify these changes we performed RT-qPCR on pools of dissected hearts, using samples biologically independent from those used for the microarray (Fig. 2f). Because the *Mesp1^Cre^* is a “Knock-In” allele in which the CRE cassette replaces the *Mesp1* coding sequence, Cre positive embryos are heterozygous for *Mesp1*. To control for any effect of *Mesp1* loss on the observed gene expression we compared three genotypes: wildtype (*Hic2^FL/FL^; Mesp1^+/+^*), Cre only (*Hic2^+/+^; Mesp1^Cre/+^*) and conditional *Hic2* mutant (*Mesp1^Cre/+^; Hic2^FL/FL^*). In wildtype embryos at E9.5, we observe strong expression of the embryonic haemoglobins, *Hbb-y* and *Hbb-bh1*, at levels of one to 2.4 times the level of GAPDH expression, with only weak expression of *Hbb-bt* (0.12 of GAPDH; Fig. 2f). In contrast, in the wildtype at E13.5, *Hbb-bt* expression has increased to 11.0 GAPDH, *Hbb-y* has increased to 11.9 GAPDH but *Hbb-bh1* expression has dropped to 0.10 GAPDH. This result indicates that primitive erythrocytes are still in circulation at this age (definitive erythrocytes never express *Hbb-y* [17]) but that they have undergone a maturational switch in gene expression from *Hbb-bh1* to *Hbb-y* [16]. The high expression of *Hbb-bt* at E13.5 indicates that definitive-lineage erythrocytes are also in circulation at this age. Cre only embryos exhibit a trend towards increased expression of embryonic haemoglobins at both timepoints, although due to high variance between samples this is not significant. The conditional *Hic2* mutant show a significant 5.6-fold increase in *Hbb-bt* expression at E9.5, from 0.12 to 0.67 GAPDH, confirming the microarray result.

To separate out the relative effects of *Hic2* and *Mesp1*, which appear to have opposing actions on erythrocyte gene expression, we calculated the change in expression between genotypes differing in only a single variable (Fig. 2g). A comparison of *Mesp1^Cre/+^* with *Mesp1^Cre/+^; Hic2^FL/FL^* shows us the relative contribution of *Hic2* because both genotypes are heterozygous for *Mesp1* (Fig. 2g, grey bars). Similarly, a comparison of *Mesp1^Cre/+^* with wildtype (*Hic2^FL/FL^*) shows us the relative contribution of reducing *Mesp1* to heterozygosity because both genotypes express normal levels of *Hic2* (Fig. 2g, blue bars). This analysis demonstrates a significant, but opposing action of *Hic2* on the definitive *Hbb-bt* and the embryonic *Hbb-bh1* such that *Hic2* acts to repress *Hbb-bt* while activating *Hbb-bh1*. *Mesp1*, in contrast, has no effect on *Hbb-bt* but shows a sub-threshold repression of *Hbb-bh1*, consistent with previous reports demonstrating that *Mesp1* acts to repress haematopoietic differentiation in favour of cardiomyocyte differentiation in the mesoderm lineage [26]. Therefore, these data suggest that *Hic2* acts to promote embryonic haemoglobin expression within MESP1 + primitive lineage erythrocytes while repressing definitive haemoglobin expression.

*Nkx2.5^Cre/+^* targets deletion in the cardiomyocyte, vascular smooth muscle and endothelial lineages [27], [28]. *Hic2^FL/FL^; Nkx2.5^Cre/+^* embryos do not show embryonic lethality and have a mild phenotype [24], and therefore we hypothesised that these embryos would not show changes in haemoglobin expression. We performed an identical RT-qPCR assay using *Nkx2.5^Cre^* in place of *Mesp1^Cre/+^* (Fig. 2g). This analysis demonstrated that loss of one copy of *Nkx2.5* had no effect on haemoglobin expression, as expected (Fig. 2g, dark grey bars). *Nkx2.5^Cre^* -mediated deletion of *Hic2* had no effect on expression of *Hbb-bt*, consistent with the absence of *Nkx2.5* in primitive-lineage yolk sac precursors (Fig. 2g, orange bars). Surprisingly however, we observed a significant reduction in *Hbb-bh1* expression in these embryos, suggesting that *Hic2* acts within NKX2.5 + precursors to promote embryonic haemoglobin expression. This result is consistent with the observation that a population of NKX2.5 + haemogenic precursors exist in the endocardium/endothelium of the outflow tract and atria at E9.5 [29], [30]. Circulating NKX2.5-derived erythrocytes initially express *Hbb-bh1*, but undergo maturational switching to express *Hbb-bt* in late gestation [29].

Thus our data are consistent with a role for *Hic2* in maturational haemoglobin switching within erythrocytes from two distinct embryological sources. *Hic2* promotes *Hbb-bh1* expression in both lineages, but represses *Hbb-bt* only in *Mesp1*-derived cells.

### 2.4 HIC2 regulates maturational isoform switching of cardiomyocytes

Creatine is an energy store used for fast production of ATP in highly active tissues such as muscle and brain. Creatine kinase, which catalyses the transfer of phosphate from creatine to ATP, exists as two isoforms in the cytosol known as muscle (CKM) and brain (CKB), and these form both homotypic and heterotypic dimers [19]. Expression is regulated both spatially and over developmental time. CKB is expressed in the early embryo in both brain and muscle; in brain it remains the only isoform expressed but in heart and skeletal muscle, expression of CKM is initiated in the foetus leading to expression first of the CKM-CKB heterodimer and later to expression of CKM alone [18].

*Ckb* was observed to be strongly expressed at both timepoints in our microarray dataset (not shown), at a level of approximately 30 × that of *Ckm* in the wildtype embryo, consistent with its known embryonic role in the heart. *Ckm* was found to be expressed at a low level at E9.5, showing a significant increase between E9.5 and E13.5. *Ckm* showed a change of + 6.78 in the *Hic2* mutant at E9.5 (Fig. 1b, c).

**Fig. 3:**
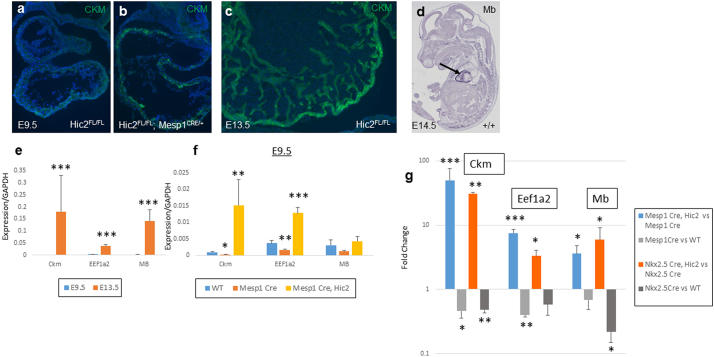
HIC2 represses foetal gene expression in cardiomyocytes. a–c. CKM Immunostaining in the wildtype (a) and mutant (b) at E9.5, and in the wildtype at e13.5 (c). CKM expression is seen in the heart tube of the E9.5 *Hic2* conditional mutant and in the ventricles wall of the E13.5 wildtype heart. d. *In situ* hybridisation to show *Mb* expression in the E14.5 heart (arrow; data obtained from Eurexpress [34]). e–g. RT-qPCR analysis of gene expression in pooled heart samples. e and f show absolute expression (normalised to GAPDH) while g shows the fold change between the genotypes indicated at E9.5. e illustrates the normal change in expression in wildtype embryos between E9.5 and E13.5 while f and g illustrate changes in mutant embryos at E9.5. *P* values indicate results of a one tailed *t*-test.

We used both RT-qPCR and immunofluorescence to verify these changes. At E9.5, expression in the wildtype heart tube is negligible (Fig. 3a, e). Strong mRNA and protein level expression is seen in the conditional mutant at E9.5 (Fig. 3b, f), the latter localised to the heart tube (Fig. 3b). At E13.5, strong CKM immunostaining is seen in the ventricles and trabeculations of the wildtype heart (Fig. 3c), while RT-qPCR assays indicate that mRNA expression increases 209-fold between E9.5 and E13.5 (Fig. 3e). We again separated the effect of *Hic2* from that of *Mesp1* within the RT-qPCR data, demonstrating that *Mesp1* has a weak but significant activator activity on *Ckm* expression (Fig. 3g; grey bars), while *Hic2* shows a strong repressor function, indicated by upregulation in the *Hic2* conditional mutant relative to *Mesp1^Cre^* only (Fig. 3g, blue bars). Analysis of *Nkx2.5^Cre^* mutants indicated that both MESP1 and NKX2.5 mediated *Hic2* deletion have a similar effect (Fig. 3g; blue and orange bars), consistent with the hypothesis that this regulation occurs in cardiomyocytes derived from MESP1 +, NKX2.5 + precursors.

Eukaryotic elongation factors such as Eef1α are a component of the translational machinery. *Eef1α* exists as two isoforms, *Eef1a1* and *Eef1a2*. While *Eef1a1* has a ubiquitous expression, *Eef1a2* expression is limited to brain, muscle and heart [31], a pattern strikingly similar to that of creatine kinase Developmental isoform switching occurs such that *Eef1a1* is expressed in embryos while *Eef1a2* is upregulated post-nataly in specific tissues [31]. The array data suggests that *Eef1a2* is expressed precociously in the *Hic2* mutant (Fig. 1c). We confirmed by RT-qPCR that *Eef1a2* expression increases 10.4-fold between E9.5 and E13.5 (Fig. 3e), and that there is a highly significant expression increase at E9.5 in both conditional *Hic2* mutants (Fig. 3f, g). *Hic2* also has an antagonistic effect to *Mesp1* and *Nkx2.5* on *Eef1a2* expression, as for *Ckm* (Fig. 3g).

Myoglobin (*Mb*) is closely related to haemoglobin but is expressed in the sarcolemma of cardiomyocytes and skeletal muscle where it transports oxygen to the mitochondria [32]. Only a single *Mb* gene exists in mammals, but maturational isoform switching has been demonstrated between the two isoforms expressed in lamprey [33]. *Mb* loss of function mice exhibit developmental delay and embryonic lethality by E11.0 [5], a phenotype consistent with a functional requirement in the foetus. Although we did not find a significant increase in the E9.5 *Hic2* mutant, our array data indicates a large 10-fold increase in wildtype *Mb* expression between E9.5 and E13.5. We therefore selected this gene for further analysis. *In situ* hybridisation data obtained from the Eurexpress database indicates specific expression in the mouse heart at E14.5 (Fig. 3d). We confirmed by RT-qPCR that *Mb* expression increases 46-fold between E9.5 and E13.5 (Fig. 3e). Consistent with the array data, we did not see a change at E9.5 in the conditional *Hic2* knockout by RT-qPCR (Fig. 3f). However, this would seem to be the result of the antagonistic effect of *Mesp1* because when we separate the effects of *Hic2* and *Mesp1* (as above) we observe a significant repressor function indicated by upregulation in the *Hic2* conditional mutant relative to *Mesp1^Cre^* only (Fig. 3g, blue bars). Deletion with *Nkx2.5^Cre^* has a similar effect (Fig. 3g, orange bars), and our data indicate that this transcription factor also has an activator effect on *Mb* expression (Fig. 3g,dark grey bars).

Thus, we have demonstrated that three developmentally-regulated genes expressed in the foetal heart are repressed by *Hic2* in the E9.5 embryo.

### 2.5 Hic2 regulates muscle-specific isoform expression in cardiomyocytes

Troponin is a component of the contractile machinery of striated muscle that acts as a calcium-sensitive switch, serving to couple motoneuron input to muscle contraction. Troponin is a complex of three unrelated proteins: Troponin T (TNNT) binds to tropomyosin, which regulates the interaction of myosin with actin, Troponin C (TNNC) binds calcium ions while Troponin I (TNNI) is an inhibitory subunit. Each exists in multiple isoforms which are regulated in a temporal and spatial manner. Three isoforms of Troponin T exist, and in the adult, these are expressed in non-overlapping domains in cardiac muscle (TNNT2), slow skeletal muscle fibres (TNNT1) and fast skeletal fibres (TNNT3) [9]. Expression of *Tnnt2* in the mouse heart begins at E7.5, the embryonic heart also transiently expresses *Tnnt1* but never expresses *Tnnt3* [35]. Foetal skeletal muscle transiently expresses low levels of *Tnnt2* together with *Tnnt1* and *Tnnt3* [35].

**Fig. 4:**
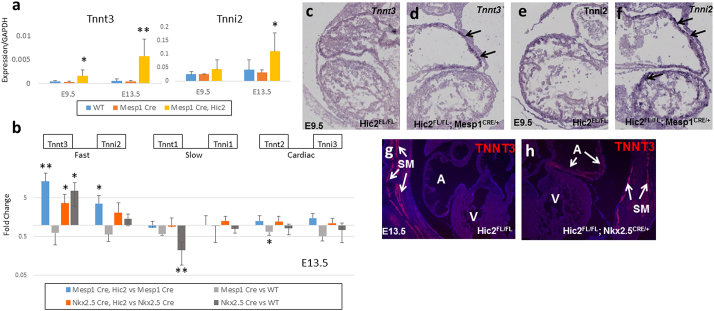
HIC2 represses cardiomyocyte expression of fast-twitch skeletal muscle troponins. a, b. RT-qPCR analysis of gene expression in pooled heart samples. a shows absolute expression (normalised to GAPDH) while b shows the fold change between the genotypes indicated at E13.5. *p* values indicate results of a one tailed *t*-test. c–f. *In situ* hybridisation showing upregulation of skeletal muscle troponins in mutant hearts at E9.5 in the *Mesp1^cre^* conditional mutant. Arrows indicate tissue showing upregulated expression. Expression of *Tnnt3* is limited to the future atrium while *Tnni2* is expressed throughout the heart tube. g–h. Immunostaining to show upregulation of TNNT3 at E13.5. In wildtype embryos (*Hic2^FL/FL^*), TNNT3 expression (red) is seen in skeletal muscle (SM) but not in the heart. In the *Nkx2.5^cre^* conditional mutant, TNNT3 expression is seen in both skeletal muscle and in the atria (A). We do not observe expression in the ventricle (V). DAPI counterstaining (blue) indicates nuclei. (For interpretation of the references to color in this figure legend, the reader is referred to the web version of this article.)

*Tnnt3* exhibits a 2.86-fold increase in the *Hic2 Mesp1^Cre^* conditional mutant at E9.5 by microarray (Fig. 1b), but unlike *Ckm*, *Eef1a2* and *Mb*, its expression did not show an increase in the E13.5 wildtype (Fig. 1c); in fact, the microarray data indicated a moderate decrease. Because *Tnnt3* is never normally expressed in the heart, we hypothesised that *Hic2* may function to regulate lineage-specific as well as maturational isoform expression. RT-qPCR confirmed the low expression level of *Tnnt3* in the wildtype heart at both E9.5 and E13.5, with no evidence for a change in expression during this time (Fig. 4a, blue bars). RT-qPCR further confirmed the ectopic expression of *Tnnt3* in the *Hic2 Mesp1^Cre^* conditional mutant, indicating a 3.6-fold expression increase at E9.5 (Fig. 4a, yellow bars). Unlike developmentally regulated genes such as *Ckm*, the effect of *Hic2* loss on *Tnnt3* expression became more pronounced with age, showing an 11.6-fold change at E13.5 (Fig. 4a, yellow bars). *In situ* hybridisation confirmed expression of *Tnnt3* in the heart tube of the *Hic2* conditional mutant at E9.5 (Fig. 4c, d). Ectopic expression appeared to be specific to the future atrium.

Troponin T genes are closely linked on the chromosome to Troponin I genes in three *Tnnt*/*Tnni* pairs [10]. Three isoforms of Troponin I exist and these are also expressed in cardiac, fast skeletal and slow skeletal muscles. The fast skeletal *Tnnt3* is linked to the fast skeletal *Tnni2* on chromosome 7 of the mouse (chromosome 10 in man) and there is evidence that these two genes are co-regulated by a common regulatory element [36], [37]. RT-qPCR showed that *Tnni2* is expressed at a level over 50 times greater than *Tnnt3* in the wildtype heart (Fig. 4a, blue bars). Expression is significantly increased in the *Hic2* conditional mutant only at E13.5, the change seen at E9.5 is not significant by RT-qPCR (Fig. 4a, yellow bars). This supports the hypothesis that dysregulation of lineage-specific isoforms becomes more pronounced with age.

We then asked whether isoforms associated with other muscle types are also dysregulated in the *Hic2* heart. We performed RT-qPCR assays at E13.5 to maximise the chance of detecting an effect and assayed the expression of all three Troponin T and all three Troponin I genes. This analysis indicated that the effect of *Hic2* loss is specific to the fast skeletal muscle troponin isoforms: we did not detect an effect on either the slow skeletal isoforms or the cardiac isoforms (Fig. 4b, blue bars). *Mesp1* did not contribute to the regulation of these genes (Fig. 4b, grey bars), although the data does demonstrate a requirement for *Mesp1* for expression of the cardiac isoform, *Tnnt2* confirming the known role of this gene in promoting the cardiac fate [26].

NKX2.5 has previously been shown to regulate *Tnnt3/Tnni2* expression [37] and therefore we asked whether HIC2 regulates *Tnnt3/Tnni2* expression within NKX2.5 + cardiomyocytes. Analysis of the *Nkx2.5^Cre^* conditional mutant confirmed the negative regulation of *Tnnt3* by NKX2.5 (Fig. 4b, dark grey bars), and also indicated NKX2.5 may positively regulate the slow skeletal troponin *Tnnt1* (which is expressed in the early embryonic heart). Expression of both *Tnnt3* and *Tnni2* are increased in the *Hic2 Nkx2.5^Cre^* conditional mutant (Fig. 4b, orange bars), but the increase is weaker than that seen in the *Mesp1^CRE^* mutant (Fig. 4b, compare blue and orange bars), perhaps indicating that NKX2.5 and HIC2 are performing partially redundant functions at this locus.

To gain a better understanding of the nature of this ectopic expression, we performed *in situ* hybridisation for *Tnnt3* (Fig. 4c,d) and *Tnni2* (Fig. 4e,f) in the *Hic2 Mesp1^Cre^* conditional mutant at E9.5. This analysis confirmed upregulation of both genes in the mutant (arrows), but indicated that while ectopic expression of *Tnni2* is seen throughout the heart tube, that of *Tnnt3* is restricted to the atria. We then performed immunostaining at E13.5 using an antibody against TNNT3 (Fig. 4g,h). A positive signal is seen to be restricted to skeletal muscle fibres in wildtype embryos (Fig. 4g), confirming specificity of the antibody. In the *Hic2 Nkx2.5^Cre^* conditional mutant we observe ectopic TNNT3 expression, which is again restricted to the atria (Fig. 4h). These data are consistent with previous reports of ectopic troponin expression in other mutants showing ectopic expression of *Tnnt3* in the *Nkx2.5* hypomorph restricted to the atria [37], while *Tnni2* can be ectopically expressed in the ventricle (for example, in the *Mesp1^Cre^* conditional *Hira* mutant [36]).

## 3 Discussion

In this work, we have described a role for the transcription factor HIC2 in regulating maturational isoform switching and muscle-specific isoform expression within the cardiovascular and circulatory systems.

### 3.1 Developmental transitions in gene expression

There are two major physiological transitions during development of the heart and circulation system, each of which is associated with isoform transitions in cardiomyocytes and erythrocytes. The better known is perhaps the major adjustment that occurs at birth, when respiration begins and oxygen is delivered to the hypoxic foetus. This results in a change in metabolism from the cytosol-based anaerobic glycolysis of the foetus to the mitochondrial-based fatty acid metabolism of the newborn [22]. This is accompanied by major changes in expression of metabolic enzymes [22], as well as isoform switching within both the cardiovascular and circulatory systems. One of the best characterised switches is the transition in myosin heavy chain expression from *Myh7* to *Myh6* that takes place within the mouse heart [38]. There is also a switch in beta haemoglobin expression from foetal to adult at this time in man (although not in mice) [4].

This switch is preceded by an earlier mid-gestation transition from an embryonic to foetal gene expression. This is clearly seen in the isoform switch from primitive to definitive (mouse)/foetal (man) haemoglobin expression, but is accompanied by other changes within cardiomyocytes. Cardiomyocyte mitochondria undergo a structural alteration at this time, changing from a fragmented, round morphology at E9.5 to an elongated, branching network at E13.5 [39]. Similarly, a reorganisation of the contractile machinery occurs at this time, resulting in generation of a mature striated cardiomyocyte morphology [39].

Single cell transcriptomic profiling would seem to broadly support this hypothesis, indicating the presence within cardiomyocytes of distinct transcriptional profiles at E9.5, E14.5 and P0 [40]. These authors were able to identify a group of genes expressed in the early embryo which are turned off by E14.5 and another set that showed the reverse relationship [40], indicating that a transition occurs at about mid gestation.

### 3.2 A developmental clock regulates maturational isoform switching

Our data implicate HIC2 in the regulation of haemoglobin isoform expression within yolk sac derived primitive lineage erythrocytes. These cells are nucleated and remain in circulation for many days after being produced, thus co-existing in the blood for a time with definitive erythrocytes [16], [17]. While definitive erythrocytes express only definitive haemoglobins, primitive lineage erythrocytes express all haemoglobin isoforms and undergo a switch in expression whilst in circulation [16], [17], [41]. We also provide evidence to suggest that HIC2 regulates haemoglobin expression in erythrocytes derived from NKX2.5 + precursors, which are most likely derived from the endocardium. There is considerable evidence that switching of haemoglobin isoform expression occurs simultaneously within erythrocytes derived from different lineages and is therefore regulated by an intrinsic “developmental clock”. This concept was proposed following the observation that cells maintained in culture [42] or transplanted into a host animal [43] switch globin expression at a time determined by the age of the donor tissue. A similar developmental clock has been proposed to coordinate postnatal switching of troponin isoforms across the cardiovascular system [44]. Our data suggests that a developmental clock may coordinate changes in the circulatory system with changes in the cardiovascular system. There are good reasons why changes in the sarcomere apparatus should be coordinated with changes in metabolic enzymes and in the oxygen delivery system. What signal HIC2 may be responding to remains to be determined, but we speculate that this may be a response to hypoxia.

### 3.3 Myocyte specific gene expression

Cardiac and skeletal muscles share a common contractile ability and utilise a set of homologous sarcomeric proteins to do this. However, cardiomyocytes are derived from lateral plate mesoderm while most skeletal myocytes are derived from the paraxial mesoderm (although facial muscle shares a common embryonic origin to cardiomyocytes, the cardiopharyngeal field [45], [46]). Thus, these cell types are analogous structures that have converged upon a common function. Although each troponin isoform is restricted to a specific muscle type in the adult, developing muscles sometimes co-express two or more isoforms [35]. It would therefore appear that a mechanism exists to suppress expression of alternative isoforms as muscles mature and that this may be disrupted in the absence of HIC2, leading to ectopic expression of the fast skeletal TNNT3 isoform.

There are parallels in this with a similar phenomenon observed during differentiation of sensory neurons. These cells are derived from two distinct embryological lineages (neural crest and neurogenic placode) but converge on a common gene expression programme. This process appears to be orchestrated by two key transcription factors, BRN3A (POU4F1) and ISL-1. Interestingly, one function of these transcription factors is to repress expression of non-sensory neuron genes, for example BRN3A represses genes normally expressed by cardiomyocytes (including *Nkx2.5*) [47] while ISL-1 does the same for genes associated with spinal motoneurons [48]. Mouse mutants exhibit activation of an ectopic programme of gene expression associated with these cell types [47], [48], [49], despite the distinct embryonic lineages from which these cell types arise.

### 3.4 Maturational and muscle-specific isoform regulation are linked

Single cell sequencing of *Nkx2.5^+/−^* cardiomyocytes at E14.5 has shown that these cells express higher levels of *Myh7* than wildtype cells [40], indicative of a delay in maturational isoform switching. The same cells also show reduced expression of *Ckm* and *Eef1a2* [40], developmentally-regulated genes that we found to be increased in the E9.5 *Hic2* heart. This is consistent with our results (Fig. 3g) and suggests that NKX2.5 may act later in development than HIC2 to promote rather than repress foetal gene expression. Ectopic expression of the fast skeletal troponins *Tnnt3* and *Tnni2* is seen in the *Nkx2.5* hypomorph mutant [37], and this is supported by our results showing increased expression in the *Nkx2.5^CRE^*. Therefore, HIC2 and NKX2.5 appear to regulate a common set of downstream genes in both maturational and muscle-specific isoform switching. It is noteworthy that both *Hic2* and *Nkx2.5* exhibit a haploinsufficient cardiac phenotype in mice [24] [50]. Another transcription factor, PROX1, has been shown to repress *Tnnt3/Tnni2* expression both in cardiomyocytes and in slow-twitch skeletal muscle [51]. While HIC2 is not expressed in skeletal muscle [24], a close homologue, HIC1, is expressed here [52] and we speculate that HIC1 may regulate lineage-specific isoform expression in skeletal muscle.

### 3.5 Mechanisms of isoform-specific expression

The Brg1/Brm-associated-factor (BAF) chromatin remodelling complex has been shown to play a critical role in regulating myosin heavy chain isoform switching. The BAF complex recruits histone deacetylase to the *Myh6* locus to repress expression, while activating *Myh7* through recruitment of poly (ADP ribose) polymerase (PARP) to a locus control region located between these two adjacent genes on the chromosome [38]. Isoform switching of haemoglobin expression also involves a locus control region, which interacts with individual globin promoters by chromatin looping [53]. A similar mechanism may operate in *Tnnt3*/*Tnni2* regulation. NKX2.5 occupies a site within the *Lsp1* gene, which is located between the paired *Tnnt3* and *Tnni2* genes, and is not expressed in cardiomyocytes [37]. This site is also bound by the histone modifier HIRA [36], and the *Hira* mouse mutant, in common with those of the histone deacetylases *Hdac1* and *Hdac2* [54], exhibits ectopic expression of *Tnnt3*/*Tnni2*. Thus, a model is emerging in which histone modifications and chromatin remodelling at an organiser region regulates transcription of isoforms located in *cis* on the chromosome. We hypothesise that the function of transcription factors such as NKX2.5 and HIC2 may be to integrate developmental signals by recruiting these modifiers to such regulatory loci. HIC2 consists of an array of DNA-binding zinc fingers at the C-terminus linked to an N-terminal BTB/POZ domain [23], which mediates transcriptional repression and acts as a protein-protein interaction domain. Little functional data exists for HIC2, but its homologue HIC1 has been shown to interact with chromatin remodelling complexes such as the NuRD (nucleosome remodelling and histone deacetylase) [55] and SWI/SNF ATP-dependent [56] chromatin remodelling complexes.

## 4 Glossary

A: atrium
BPM: beats per minute
BTB/POZ: a protein domain named after proteins in which it is found (Bric-a-brac, Tramtrack, Broad-Complex/Poxviruses and Zinc fingers)
*Ckb*: creatine kinase, brain
*Ckm*: creatine kinase, muscle
CRE: CRE recombinase (causes REcombination), a recombinase enzyme derived from the P1 bacteriophage which recognises the LoxP site
E: erythrocyte
E9.5: Embryonic day 9.5. By convention, day E0.5 is midday on the day following conception.
*Eef1a*: eukaryotic elongation factor 1a
FL: floxed allele, in which LoxP sites flank one or more exons.
GT: genetrap allele, in which a cassette consisting of a splice acceptor site, a LacZ coding sequence and a polyadenylation signal are randomly inserted into the genome. If inserted into a coding sequence may cause a loss of function mutation.
*Hbb-bh*: haemoglobin Z, beta-like embryonic chain
*Hbb-bs*: haemoglobin, beta adult s chain
*Hbb-bt*: haemoglobin, beta adult t chain
*Hbb-y*: haemoglobin Y, beta-like embryonic chain
*Hic2*: hypermethylated in Cancer 2
HT: heart tube
KO: knockout, or loss-of-function allele
LOXP: short DNA sequence recognised by the CRE enzyme
*Mb*: myoglobin
*Mesp1*: mesoderm posterior 1
NuRD: nucleosome remodelling and histone deacetylase complex
P0: postnatal day 0, or the day of birth
SM: skeletal muscle
SWI/SNF: SWItch/Sucrose Non-Fermentable, a chromatin remodelling complex
TNNC: troponin C, calcium
TNNI: troponin I, inhibitory
TNNT: troponin T, tropomyosin
V: ventricle
WT: wildtype allele
X-GAL: X-galactosidase staining, indicates expression of LacZ.

## 5 Methods

### 5.1 Mouse genetics and breeding

Mouse lines used in this study have been previously described [24].

Hic2^GT^:Hic2^Gt(RRN127)Byg^; MGI:4329590

Hic2^FL^:Hic2^Gt(E225A08)Wrst^; MGI:3919233

Nkx2.5^CRE^:Nkx2–5^tm1(cre)Rjs^; MGI:2654594

Mesp1^CRE^:Mesp1^tm2(cre)Ysa^; MGI:2176467

Throughout the paper, mice carrying the Hic2^FL^ allele in the absence of a Cre allele are considered wildtype. All mouse procedures were carried out in accordance with UK Home Office regulations.

Timed matings were performed and pregnant dams harvested by cervical dislocation.

### 5.2 RNA extraction from heart

Whole heart containing circulating erythrocytes was dissected from embryos lacking a phenotype at both E9.5 and E13.5 and stored in RNA-Later (Ambion). RNA was prepared using the RNA mini kit (Qiagen). Three genotypes were analysed for each conditional mutant: CKO (Cre/+; Hic2^FL/FL^), CRE (Cre/+; Hic2^+/+^) and WT (+/+;Hic2^FL/FL^or +/+;Hic2^FL/+^). Two knock-in CRE lines were used in this study: Mesp1^Cre^ and Nkx2.5^Cre^. 4–6 hearts were pooled per sample and six independent biological replicates (each itself a pooled sample) were performed for each genotype group, three were used for microarray analysis and an independent three pools used for subsequent qPCR analysis.

### 5.3 Microarray analysis

Single stranded cDNA was prepared using the Ambion WT Expression kit (Ambion) and this was hydrolysed and labelled using the Affymetrix genechip terminal labelling and hydrolyzing kit (Affymetrix, Santa Clara, USA). Probes were hybridised to the Mouse Exon 1.0 ST whole transcript array genechip (Affymetrix) at UCL Genomics.

Data were analysed using the core gene predictions of the Affymetrix Gene Expression Console and normalised using the RMA algorithm. Data were pre-filtered to select for those genes showing an expression value > 100 in the upregulated condition and to remove probes for which no annotation exists. We then performed a two tailed *t*-test to test the null hypothesis of no difference between the wildtype and knockout conditions. This gave 174 upregulated and 457 downregulated genes. Applying an arbitrary threshold of mean fold change > 1.5 across 3 replicates reduced this number to give a final list of 34/29 upregulated/downregulated changed genes.

CEL and CHP files of these microarray data have been submitted to the Gene Expression Omnibus, accession numbers: E13.5 data GSE56430 and E9.5 data GSE100125.

### 5.4 qRT-PCR

RNA was reverse transcribed using the Quantitect Reverse Transcription kit (Qiagen). qPCR was performed using SYBR Green technology on a Step One machine (Applied Biosystems). Expression was measured relative to that of GAPDH and significance assessed by performing a one or two tailed t-test on the Ct values. Graphs show mean ± standard deviation.

### 5.5 *In situ* hybridisation

*In situ* hybridisation was performed on cryosections with DIG labelled probes using standard techniques. Briefly, tissue was permeabilised with a 10 minute digestion in 10 μg/ml proteinase K, hybridised at 55 °C with 1 ng/ml DIG-RNA probe and probe detected with a secondary alkaline phosphatase sheep anti DIG polycolonal antibody (Roche). Matched control and mutant sections were collected and processed on the same slide to ensure that hybridisation and colour development conditions were equal.

### 5.6 Immunofluorescence

Commercial goat anti CKM (Santa Cruz 15,164) and rabbit anti TNNT3 (Sigma HPA 037810) polyclonal antibodies were used. A citrate antigen retrieval step was used for the TNNT3 antibody. Alexa-conjugated secondary antibodies were used (Life Technologies, Carlsbad USA). Sections were mounted in Vectashield medium containing DAPI (Vector laboratories).

### 5.7 X-gal staining

β-Galactosidase staining was performed using standard methods.

## Funding

This project was funded by the British Heart Foundation (PG/09/065/27893).

## Disclosure and conflicts of interest

None
