# Corrigendum to “HIC2 regulates isoform switching during maturation of the cardiovascular system” [Journal of Molecular and Cellular Cardiology volume 114 (2018) P29-37/ https://doi.org/10.1016/j.yjmcc.2017.10.007]

**DOI:** 10.1016/j.yjmcc.2023.07.010

**Published:** 2023-10

**Authors:** Iain M. Dykes, Kelly Lammerts van Bueren, Peter J. Scambler

**Affiliations:** a Liverpool John Moores University, Byrom St, Liverpool L3 3AF, United Kingdom; b Institute of Child Health, University College London, 30 Guilford St, London WC1N 1EH, United Kingdom

The authors regret that there was an error in the labelling of the axes shown in the graphs in Figs. 1b and 1c. In both cases, the x-axis label should read E9.5 KO / E9.5 WT (Log2) and not E9.5 WT / E9.5 KO WT (Log2) as originally stated.

The email address for the corresponding author has been changed to i.m.dykes@ljmu.ac.uk.

The authors would like to apologise for any inconvenience caused.

